# Implantable Devices for the Treatment of Breast Cancer

**DOI:** 10.3390/jnt3010003

**Published:** 2022-02-09

**Authors:** Mohammad Mohtasim Hamid Pial, Asahi Tomitaka, Nezih Pala, Upal Roy

**Affiliations:** 1 Department of Electrical and Computer Engineering, Florida International University, Miami, FL 33174, USA; 2 Department of Immunology and Nano-Medicine, Herbert Wertheim College of Medicine, Florida International University, Miami, FL 33199, USA; 3 Department of Computer Science, University of Houston-Victoria, Victoria, TX 77901, USA; 4 Department of Health and Biomedical Sciences, University of Texas Rio Grande Valley, Edinburg, TX 78539, USA

**Keywords:** implantable drug delivery devices, localized therapy, chemotherapy, biopolymer, nanocomposite materials

## Abstract

Breast cancer is one of the leading causes of death in the female population worldwide. Standard treatments such as chemotherapy show noticeable results. However, along with killing cancer cells, it causes systemic toxicity and apoptosis of the nearby healthy cells, therefore patients must endure side effects during the treatment process. Implantable drug delivery devices that enhance therapeutic efficacy by allowing localized therapy with programmed or controlled drug release can overcome the shortcomings of conventional treatments. An implantable device can be composed of biopolymer materials, nanocomposite materials, or a combination of both. This review summarizes the recent research and current state-of-the art in these types of implantable devices and gives perspective for future directions.

## 1. Introduction

One of the most common types of cancer is breast cancer, which primarily affects the female population, and its mortality rate is next to lung cancer for women [1,2]. The breast undergoes a mutation, which causes tumor growth. In some cases, even after removing cancerogenic tissue/tumor, there is still some cancer cell residue left at the affected area, which can cause a local recurrence of breast cancer or metastasis [2–4]. Breast cancer cells might affect other organs, but it usually happens at a much later stage. In the United States, it was estimated that there were 268,600 breast cancer cases among women in 2019, and the mortality rate was approximately 15.54% [5–7]. The estimation of breast cancer cases in women increased to 276,480 and the mortality rate in women was 42,170 in 2020 [8]. The estimation by the National Cancer Institute’s Surveillance, Epidemiology, and End Results (SEER) Program states that in 2021, 281,550 women in the United States would be diagnosed with breast cancer and 43,600 would die of the disease [9].

The traditional approaches for cancer treatment such as chemotherapy, hormonal therapy, and immunotherapy are systemic treatments, which deliver therapeutic agents to the entire body [10,11]. In addition to this, surgery is considered the main approach in early stages of breast cancer, and it is a nonaggressive procedure. Despite the noticeable results, conventional cancer treatments cause systemic toxicity, including apoptosis of healthy cells [11,12]. The drugs used for the conventional approaches have low solubility and rapid metabolism, leading to poor pharmacokinetics [1,11,13]. Another major drawback of conventional treatment is its inability to deliver the specific drug to the targeted area, and they remain there for a prolonged period [1], which leads to severe side effects and increased probability of recurrence [1,14].

Local therapy addresses these issues by directly delivering drugs to the affected area via implantable devices or drug delivery nanocarriers [10]. Nanomaterials have attracted significant attention due to their immense potential for applications in cancer treatment. Nanomaterials have unique properties including modifiable surface chemistry due to their large surface area-to-volume ratio. The surface can be functionalized with bioactive molecules for targeted drug delivery for cancer treatment, thus increasing the overall efficacy of the therapy [1,15–20]. Targeted drug delivery is typically achieved by the following two approaches: passive targeting and active targeting [21–23]. The pathophysiological difference between normal and tumor tissues enables the nanoparticles to penetrate the tumor site. This type of targeting process is known as passive targeting [1,22,23]. Active targeting is achieved by the conjugation of high-affinity targeting ligands to the surface of the nanocarrier [24,25].

Implantable drug delivery devices enhance the therapeutic efficacy of cancer treatment by achieving local administration of drugs to the tumor site as well as by protecting the loaded drugs from degradation or clearance until they are released [11]. They also reduce the overall drug concentration in blood circulation by enabling precise spatial control, prevent damage to healthy cells, and increase the overall survival rate [11]. Moreover, combination treatments such as chemotherapy and gene therapy can be applied using a single implantable device [26–28]. One way to control drug release from implantable drug delivery devices is to use passive mechanisms in polymer systems. The release method is governed by diffusion, drug carrier affinity, polymer degradation, or a combination of these mechanisms [11,29]. This temporal control over the drug release profile helps maintain therapeutic concentrations in the affected area over a longer period of time [12]. The second way to control the drug release is to use internal stimuli (e.g., body temperature, pH) or external stimuli (e.g., temperature, electromagnetic waves, ultrasound, visible light, infrared (IR) light, and near-infrared (NIR) light) [14,29]. These external stimuli cause rapid changes in dimensions or physical properties of implantable systems, which lead to the release of therapeutic agents [11,14,29].

Work on various types of implantable devices to treat different types of cancers has been published. Wright et al. implemented an osmotic system-based implantable device to deliver the GnRH agonist, leuprolide, to humans for the palliative therapy of advanced prostate cancer [30]. The work presented in [31] utilized β-lapachone containing polymer implants (millirods) to treat prostate tumors. A polymer delivery device can control release, which increases the antitumor efficacy. HUVEC (human umbilical vein endothelial cells) and PC3 (prostate cancer) cells were used to investigate the biological activity of docetaxel (DTX) released from a magnetically controlled drug delivery MEMS device [32]. An in vitro study to decrease local recurrences in a murine model of Lewis lung carcinoma using biocompatible poly(glycerol monostearate-co-e-caprolactone) polymer film-loaded paclitaxel (PTX) implants was conducted by Liu et al. [33]. Wolinsky et al. utilized a tunable drug-eluting polymeric delivery platform made up of poly(glycerol monostearate-co-ɛ-caprolactone) films loaded with anticancer agent 10-hydroxycamptothecin (HCPT) [34]. The film formed a flexible composite when applied to a collagen-based scaffold clinically indicated for the mechanical reinforcement of lung tissue. The composite releases drugs over seven weeks, thereby preventing the local growth and establishment of Lewis lung carcinoma tumors in vivo. The work by Ramachandran et al. applied polyester nanofibers of PLGA–PLA–PCL blends, which were electrospun together to form a flexible three-dimensional (3D) composite nanofiber implant for controlled delivery of anticancer drug temozolomide to an orthotopic brain tumor for one month. Prolonged drug release had control in tumor growth and prohibited tumor recurrence in orthotopic brain tumor models, wherein >85% of wafer-implanted animals had a survival rate of 3 months [35]. There were several clinical trials where some types of implantable devices were used for cancer treatment. M. Westphal et al. conducted a phase III study on 240 patients to confirm that biodegradable 1,3-bis(2-chloroethyl)-1-nitrosourea (BCNU) wafers (Gliadel wafers) prolong survival in patients with recurrent glioblastoma multiforme [36]. Gliadel wafers were approved in 1996 by the FDA as an adjunct to surgery in patients with recurrent glioblastoma multiforme. The product is now approved in 23 other countries for this indication and in Canada for recurrent and newly diagnosed malignant glioma. Another clinical investigation was carried out on 100 participants for ultrasound-guided implant radiation therapy to treat prostate cancer [37]. However, no clinical trials of implantable devices for breast cancer treatment were obtained during the literature survey for this article. All the work explored in this manuscript is based on animal models. The list of implantable devices for various cancer treatments is given in Table 1.

This review paper focuses on the reported efforts in the field of implantable devices for breast cancer treatment. The work reported here focuses on biodegradable, biopolymer nanocomposite, nonbiodegradable, and nanocomposite-based implantable devices. Injectable hydrogel is also used for breast cancer treatment as it can have controlled drug release, helps with wound regeneration, and is considered as a type of implantable devices in this article. The paper aimed to summarize the materials and methods used in the recent implantable devices as well as their effectiveness in treating breast cancer.

## 2. Breast Cancer and Typical Treatments

Surgical procedures are a standard treatment for any abnormal growth or tumor in the human body. Two standard surgical treatments for breast cancer are mastectomy and lumpectomy. In the lumpectomy procedure, the tumor and some surrounding normal breast tissues are removed, keeping the rest intact. It is considered as a breast-conserving surgery. Generally, radiation therapy is needed to destroy any remaining cancer cells to prevent recurrence. On the contrary, mastectomy is a surgical procedure that removes the entire breast. By removing the entire breast, the probability of recurrence decreases. Moreover, the patient who undergoes mastectomy usually does not require additional surgery or radiation therapy. Early detection and removal of the cancerous tumor prevent cancer metastases to other body sites, which leads to favorable prognosis. In addition to surgery and tumor removal, other treatments such as chemotherapy, hormone therapy, and radiation therapy are carried out to increase the effectiveness of the overall treatment [1].

Chemotherapy is a treatment process that administers antineoplastic drugs orally or intravenously. The drugs work by halting cancer cell cycle progression and inducing cellular apoptosis. One of the most common drugs used for chemotherapy is doxorubicin (DOX). DOX is an anthracycline type of chemotherapy drug which blocks an enzyme called topoisomerase 2 to stop the growth of cancer cells [39,40]. Other widely used chemotherapy agents include PTX and DTX. Two approaches are followed when chemotherapy is used in breast cancer treatment: neoadjuvant or adjuvant chemotherapy. Neoadjuvant therapies shrink the tumor size before the primary treatment, which is usually surgery [41]. Therefore, neoadjuvant chemotherapy is often used for cancer tumors that are too large to be removed by incision. Adjuvant therapies are applied after removing the tumor to prevent cancer recurrence [42].

Hormone therapy, which is also known as endocrine therapy, uses hormones to stop or slow down the growth of cancerous cells [1]. Estrogen and progesterone are hormones that can promote the growth of some breast cancer cells based on hormone receptors [43–45]. There are two types of hormone therapy drugs: blockers and inhibitors. Hormone inhibitor drugs target breast cancer cells with hormone receptors and reduce hormone production, which causes the tumor size to shrink. In contrast, hormone blocker drugs block the estrogen-shaped openings in the cells and thereby prevent the growth of estrogen-fueled cancers cells.

Based on the hormone receptor status, breast cancer can be clinically categorized into three types: hormone receptor-positive/ERBB2-negative, ERBB2-positive, and triple-negative breast cancers [1,46]. Depending on the types of breast cancer, various kinds of treatment are applied. Hormone receptor-positive are estrogen receptor-positive (ER+)/progesterone receptor-positive (PR+) breast cancers; they accounts for 85% of all breast cancer cases [6]. They can be further classified into two subtypes: luminal A and luminal B. Luminal A tumors tend to be ER+ and PR+ and HER2-negative (HER2−). Luminal B tumors tend to be ER+ and/or PR+ and HER2+ (or HER2− with high Ki67) [6]. Ki67 is a protein associated with cell proliferation [47]. A high value of Ki67 indicates rapid growth of the tumor. The treatment for this type of cancer is endocrine therapy, with few cases receiving chemotherapy [1,46]. HER2-positive breast cancer is a breast cancer that tests positive for human epidermal growth factor receptor 2 (HER2). On average, 20% of all breast cancers are HER2+ [1,46]. This type of breast cancer can be treated using anti-HER2 drugs. Among all the types of breast cancer, triple-negative breast cancer has the highest probability for recurrence and is the hardest to treat [1,46]. It is a type of breast cancer that does not express the estrogen receptor, progesterone receptor, or HER2 receptor genes. Due to this lack of hormone receptor expression, the treatment is complex, and it accounts for 15% of the breast cancer cases [6]. Since this type of breast cancer does not overexpress receptors, targeting therapies are not an option. Conventionally, it is treated with a combination of surgery, radiation therapy, and chemotherapy [6].

A high radiation dose is used for radiation therapy to kill cancer cells and shrink tumors [1,48]. Radiotherapy is usually applied after breast conservation surgery and post-mastectomy [49]. Follow-up radiation therapy after breast-conserving surgery decreases the recurrence rate by 50% and the mortality rate by one-sixth [49]. Therefore, patients suffering from early and locally advanced breast cancer have a higher survival rate after radiation therapy. However, radiation therapy also has some drawbacks, including decreased sensation in the breast tissue and skin problems in the treated area. It was observed that the skin may become moist at the end of treatment [48]. Internal radiation therapy with a stable source is the typical radiotherapy treatment for breast cancer. The treatment is called brachytherapy, and the radiation is delivered to the body or near the cancer area in capsules or liquid [1].

Even though the conventional treatments mentioned above have significant outcomes in treating breast cancer, there are still major drawbacks that are needed to be overcome. Conventional procedures utilize the systematic drug delivery method which can damage healthy organs, tissues, and cells along with the infected tumor. The application of local drug delivery for targeted treatment by using implantable devices can be one method to overcome these shortcomings.

## 3. Implantable Device for Breast Cancer Treatment

### 3.1. Biopolymer-Based Implantable Devices

Polymeric materials which can either be chemically derived from biological materials or entirely biosynthesized by living organisms are known as biopolymers. Recently, considerable research has been carried out on biopolymer-based implantable drug delivery devices. Both biodegradable and nonbiodegradable biopolymer materials are utilized in these implants. Notable advantage of biopolymeric implantable drug delivery devices are their biocompatibility, tunability, and ability to deliver multiple drugs. Biodegradable biopolymers degrade into substances that can be naturally absorbed/excreted by the body [11]. Nonbiodegradable implants can be removed surgically after the treatment procedure is completed. Moreover, the adverse reactions of the human body to these biopolymers in most cases are mild. Biopolymers enable multiscale control over the release kinetics of the loaded therapeutic drugs [11]. One way to attain this is to incorporate them with nanoparticles which enable stimulus-controlled drug release. The incorporation of synthetic polymers causes structural changes allowing implants to carry multiple drugs and having different release mechanisms for these drugs. Biopolymeric implantable drug delivery devices are classified under two distinct categories: preformed systems and in situ formed implants [11,50]. Preformed implants have their structure fixed before implantation. The shape and surface area of the implant have a significant role in the drug release profile and polymer degradation [11,50]. In situ formed implants are considered as a less invasive treatment method. This category of implants comes in the form of a solution or suspension of the matrix and active agents [50]. The solution is injected into the target and solidified into an “implant” in response to a stimulus (usually, the pH or body temperature near the affected area) [50,51]. The drug is released through a process of diffusion as the gel slowly degrades in the body.

#### 3.1.1. Biodegradable Implantable Devices

Chitosan nanoparticles have a high potential as a passive targeted drug delivery carrier since they exhibit high efficiency for sustained drug release [52–54]. A. Kefayat et al. incorporated poly(lactic-co-glycolic) acid (PLGA), a biodegradable polymer, with chitosan-loaded doxorubicin (CS-DOX) in various ratios (PLGA:CS-DOX = 1:1, 2:1, and 4:1) [55]. The implant’s drug release profile depends on the initial drug diffusion in the chitosan nanoparticles followed by erosion of the PLGA matrix. The in vitro release of DOX from the implants follows two phases: a burst release and a sustained release. In contrast to the ratio of 2:1 and 4:1, the release profile of PLGA:CS-DOX (1:1) exhibited sustained drug release as 39% of DOX were discharged in the first phase, and the cumulative amount of 60% in the second phase. Studies were conducted with 4T1 tumor-bearing BALB/c mice treated with PLGA, PLGA/CS implants (subcutaneous implantation), and DOX. Female BALB/c mice (age: 6–8 weeks, weight: 25 ± 2 g) were acclimated for at least one week before the start of the study and maintained throughout at standard conditions: 24 ± 2 °C temperature, 50 ± 10% relative humidity, and a 12 h light/12 h dark cycle. All the mice were fed sterilized standard mouse chow and provided with water ad libitum. The slow release rate of PLGA/CS allowed longer retention of the drugs in the tumor and increased the therapeutic efficacy. PLGA/CS-DOX showed a significant antitumor effect and inhibition of metastatic nodules’ formation at lungs which was validated by the increase in survival time to 120 days in the mice groups treated with the implants. Additionally, the group had the smallest 4T1 breast tumor growth, leading to a higher survival rate.

**Figure 1.**
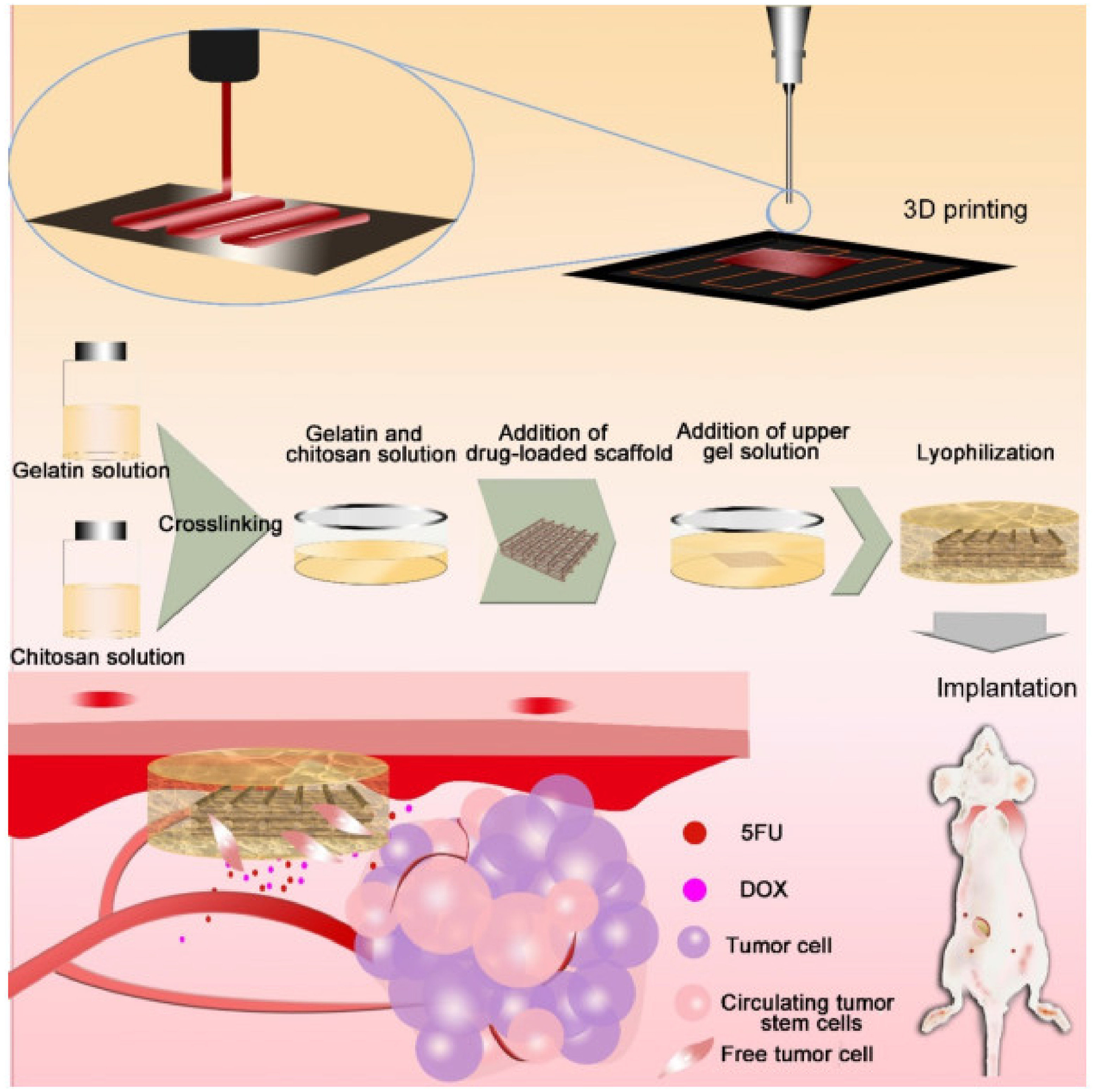
Fabrication of an intelligent scaffold. Hydrodynamic jet 3D printing was used to print a drug-loaded scaffolds. They were then sandwiched between a gelatin–chitosan gel. The scaffolds were implanted in vivo to absorb hemorrhage and cell residues after surgery and inhibit cancer cells and circulating tumor cells. Reprinted under the Creative Commons Attribution 4.0 International license from [56].

Shi et al. worked on an intelligent 3D-PLGA, gelatin, and chitosan scaffold loaded with anticancer drugs 5-FU and DOX for simultaneous cancer treatment and wound healing [56]. The pH-responsive Schiff base complexes formed by gelatin and chitosan and the degradation of PLGA in an acidic environment enabled a controlled release of the drugs. Furthermore, the implant proposed here can reduce the risk of cancer metastasis and recurrence by local hemostasis and absorption of free cells. Hemorrhage and cell residues are absorbed by the scaffolds after surgery, promoting wound healing. In order to ensure pH responsiveness, the drug-loaded PLGA–DOX–5-FU (PD5) scaffolds were sandwiched between a gelatin–chitosan (GC) gel to fabricate an intelligent scaffold (IS) (Figure 1). The blood clotting index (BCI) of the GC scaffolds was 7.5-fold higher than that of the IS, indicating a good clotting ability of IS; it also has a good absorption capacity for deionized water and blood, which was 1.2-fold higher than in the GC group. Both of these properties help with wound healing. A 30-day experiment in vivo with MDA-MB-231 cells treated with DOX (0.94 μM) and 5-FU (0.31 mM) hybrid solutions, PD5 and IS, respectively, was conducted to investigate the antitumor efficacy. The in vivo anti-tumor study was carried out on 24 nude female BALB/C mice divided into four groups. The group treated with IS had a slower tumor growth rate, lower recurrence rate, and higher survival rate compared to other groups. The average tumor volume in control, DOX + 5-FU, GC, and IS groups were 265.47 mm^3^, 164.39 mm^3^, 90.93 mm^3^, and 55.07 mm^3^ respectively. No metastases were observed in both IS and PD5 groups. The IS group had a better therapeutic effect than the PD5 group, indicating that the drug-loaded scaffolds inhibited tumor growth and reduced the risk of distal metastasis. The IS group also had the lowest recurrence rate of 61.33%. Additionally, no significant changes in body weight were observed for the IS group, and no damage was observed in the liver, spleen, and kidney tissues.

**Figure 2.**
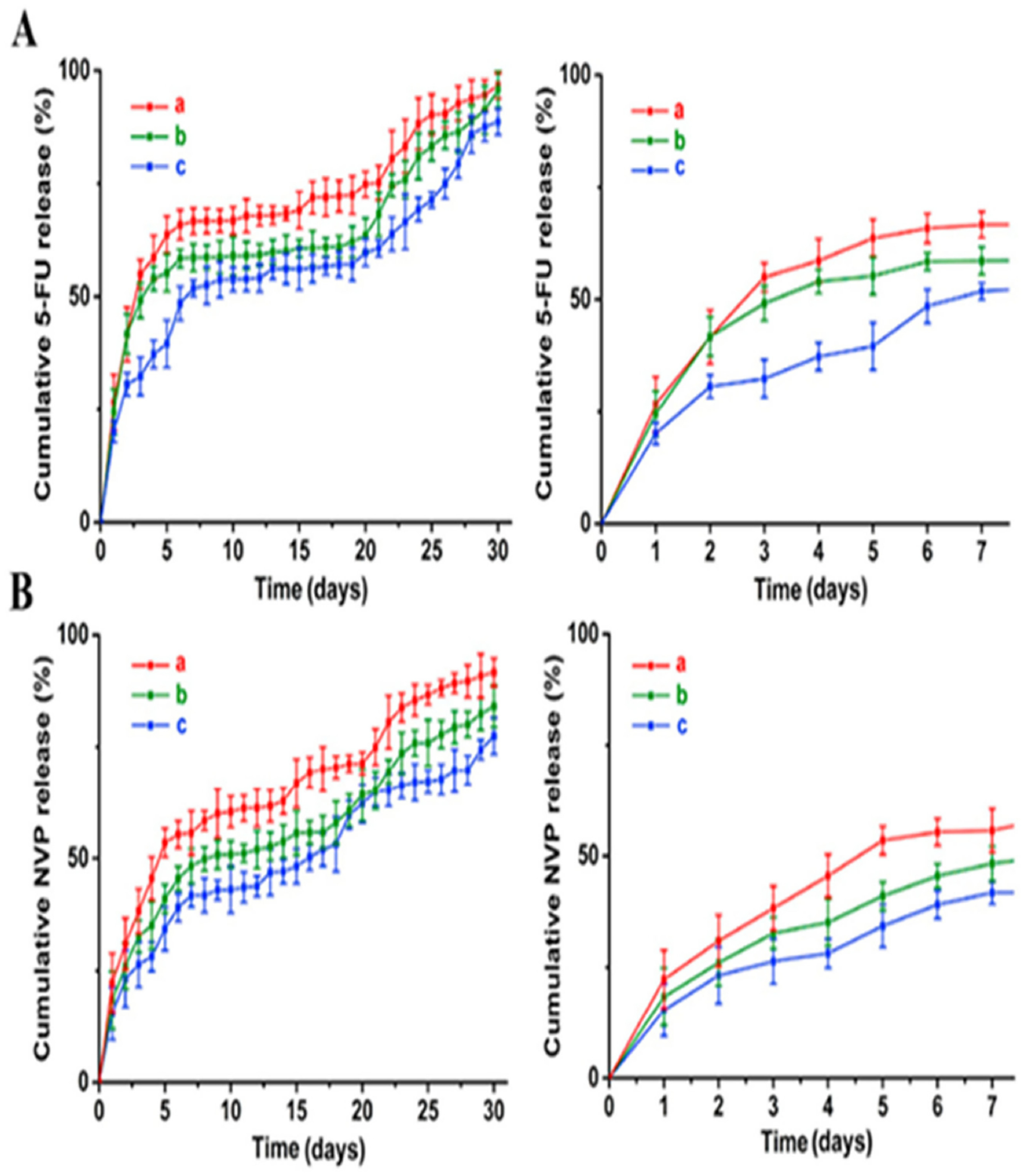
Release profiles of (**A**) 5-FU and (**B**) NVP-BEZ235 in PFN scaffolds with varying aperture sizes, (a) 150 μm, (b) 100 μm, (c) 50 μm, over 7 and 30 days. Reprinted with permission from ref. [57]. Copyright 2019 Elseveir.

Scaffolds-based drug delivery devices for orthotopic breast cancer therapy, which can suppress breast tumor growth and reduce pulmonary metastasis using combination chemotherapy, have also been explored [57]. The PLGA scaffolds having aperture sizes of 50 μm, 100 μm, and 150 μm fabricated using 3D printing were immobilized with 5-fluorouracil (5-FU) and NVP-BEZ235; the former, 5-FU, suppresses the synthesis of nucleic acids and induces apoptosis in cancer cells, whereas the latter, NVP-BEZ235, is a reversible PI3K/mTOR inhibitor that has been shown to significantly decrease tumor growth. Combination chemotherapeutic drug treatment induces tumor suppression based on the p53 upregulated modulator of apoptosis. PLGA degradation allows for a long-term drug release, whereas scaffold aperture size controls the release rate. Approximately half of both the drugs was released within the first week, followed by a slow and quick release stage, respectively (Figure 2). The scaffolds with an aperture size of 150 μm exhibited a higher drug release rate than others, as more than 90% of 5-FU and NVP-BEZ235 was released in the first month. Nude female BALB/c mice (4 weeks old), which were kept at pathogen-free conditions, were used for the breast cancer model. An MDA-MB-231 cell suspension (0.2 mL, 5 × 10^7^ cells/mL) was injected into the mice to establish an orthotopic breast tumor model. The cell viability of MDA-MB-231 incubated with a PFN scaffold was below 40% after seven days, lower than that of the cells treated with free 5-FU, NVP-BEZ235, and dual drugs. A 37.83% apoptosis was observed on the MDA-MB-231 cells incubated with PFN scaffolds, which was higher than that of the control (2.37%), 5-FU (15.19%), NVP-BEZ235 (7.66%), and dual drugs (21.47%) groups. The results verify that PFN scaffolds had the highest antitumor efficiency among the tested groups. The hemolysis rate for the PFN scaffolds reached a maximum of 1.21%, much smaller than the safe limits (5%). The antitumor effects of PFN scaffolds in MDA-MB-231 tumor-bearing nude Balb/c mice were evaluated following a four-week treatment. The mean tumor volumes were less than 600 mm^3^ for the mice treated with scaffold implants. H&E staining results suggest that the PFN scaffolds reduced the number of metastatic foci in lungs, likely reflecting better inhibition of primary tumor growth and the increase in the survival rate.

**Figure 3.**
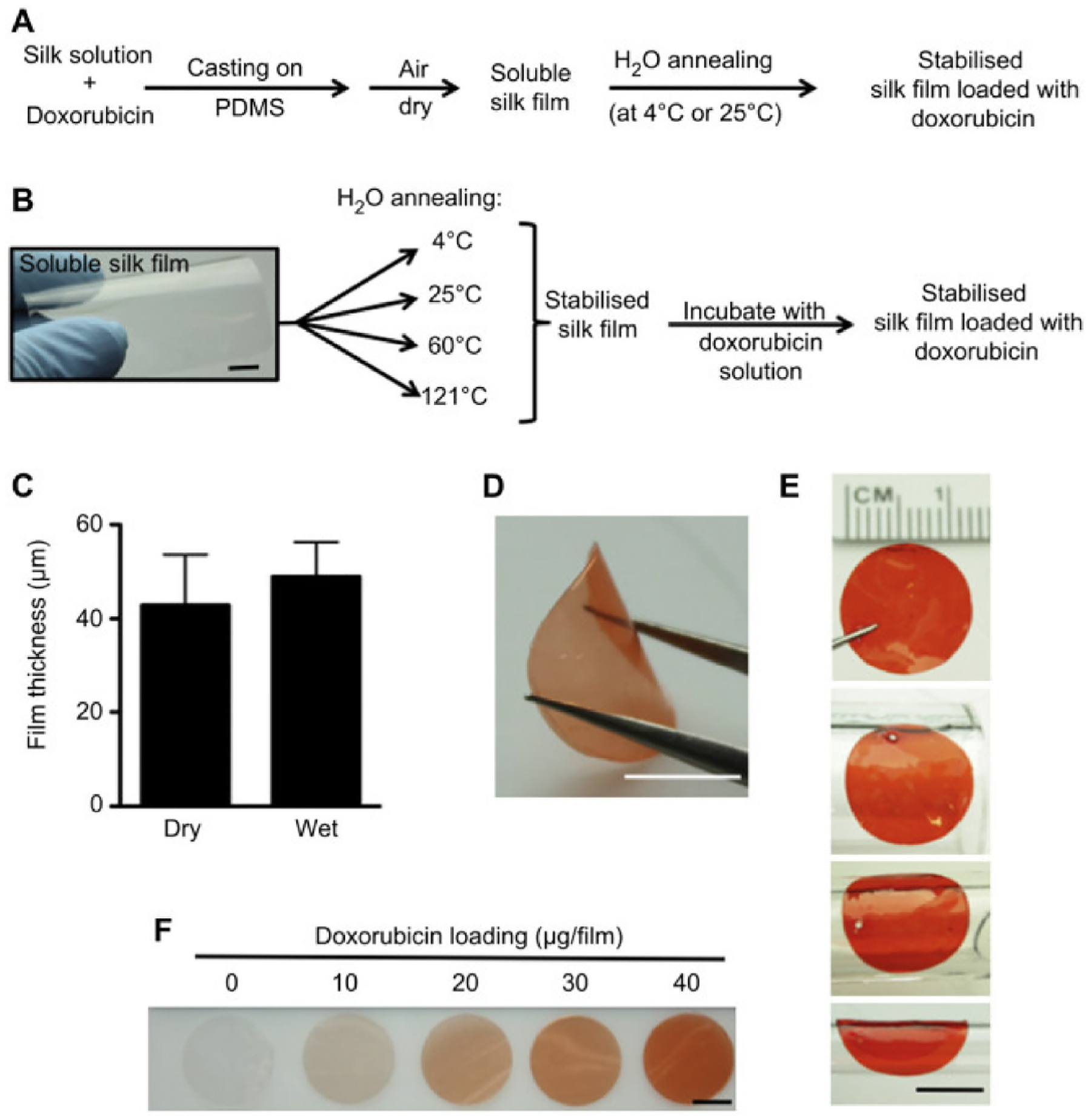
Preparation of doxorubicin (DOX)-loaded silk films. (**A**) Techniques to synthesize DOX-loaded films that are water-soluble or (**B**) water-insoluble with variable beta-sheet content. (**C**) Film thickness of dry and hydrated silk films. (**D**) Doxorubicin-loaded silk films in the dry state (scale bar, 7 mm). (**E**) Hydrated films following surface contours (scale bar, 7 mm). (**F**) Various concentration ranges of DOX loaded in silk films. Reprinted with permission from ref. [58]. Copyright 2012 Elseveir. from different

Slow degradation rate and biocompatibility make silk an excellent drug carrier. Seib et al. applied DOX-loaded silk films having different crystallinity contents or beta-sheets (Figure 3) which were fabricated using water vapor annealing for breast cancer treatment [58]. The adenocarcinoma breast cancer cell line MDA-MB-231 was used for the experiment here and maintained in a humidified atmosphere of 5% CO_2_ at 37 °C and was routinely subcultured every 2–3 days. MDA-MB-231 cells were grown in RPMI 1640 supplemented with 10% (*v*/*v*) FBS. Circular silk film was directly applied to tumors, and the release rate of DOX was controlled by manipulating silk crystallinity. Beta-sheet contents accounted for 14%, 30%, 50%, and 57% of the secondary structure after water vapor annealing at 4 °C, 25 °C, 60 °C, and 121 °C, respectively. Two methods were applied for loading the drug: (1) doping the silk solution with DOX before casting, which produced a soluble silk film, and (2) using stabilized silk films that were soaked in DOX solutions. A four-week study indicated that as the beta-sheets content increased, the cumulative DOX release decreased. The cumulative DOX released from stabilized silk film after four weeks was 56% and 38% for the silk films annealed at 4 °C and 121 °C, respectively. The breast cancer cell (MDA-MB-231) viability and the tumor weight were the lowest when treated with stabilized silk followed by a soluble silk film and free diffusible DOX.

Ding et al. developed DTX-loaded poly-D,L-lactide (PDLLA) nanofibers with 5, 10, and 20 wt% DTX to evaluate its therapeutic effect in preventing local breast cancer recurrence [59]. DTX has a higher affinity to b-tubulin and resides inside cells for a longer period than PTX and it showed a favorable pharmacological profile [60]. The mass ratio of DTX and PDLLA controlled the release rate [59]. In the first 12 h, an initial burst release was observed, followed by a slow release ratio. Approximately 23.3%, 25.3%, and 29.6% of DTX was released from DTX/PDLLA nanofibers containing 5, 10, and 20 wt% DTX within 24 days, respectively. Female BALB/c mice, weighing 20 ± 2 g, were used for this study; they were housed in a controlled temperature of 20–22 °C, relative humidity of 50–60%, and 12 h light–dark cycle separated by sex. In vitro release study showed that the 4T1 breast cancer cells’ viability decreased from 50% on day 1 to 10% on day 3 when incubated with DTX/PDLLA scaffolds containing 20 wt% DTX, which was considerably lower than the cells incubated with other samples. A long-term drug release was achieved, maximizing tumor toxicity while minimizing systemic toxicity. The recurrence time in the mice treated with DTX/PDLLA nanofibers was delayed to 15 days after tumor resection, and only two out of 12 mice developed LRR. LRR rates of the mice treated with local administration of DTX, blank PDLLA nanofibers, intravenous injection of DTX, and the control group were 77.8%, 88.9%, 75%, and 100%, with a median time of 13, 11.7, 11.9, and 11 days, respectively.

#### 3.1.2. Nonbiodegradable Implantable Devices

**Figure 4.**
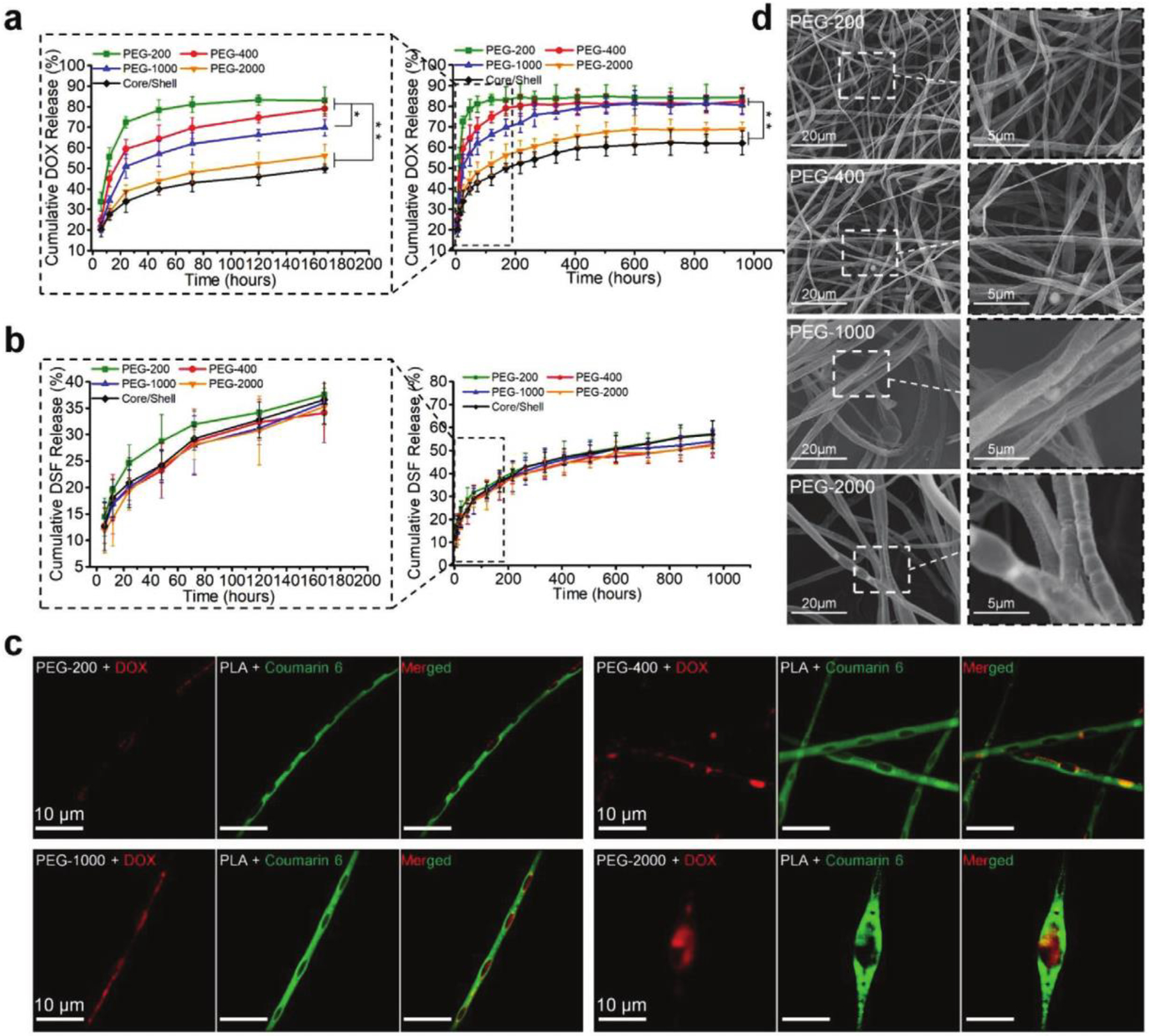
(**a**) Release behaviors of DOX-HCl. (**b**) Release behaviors of disulfiram (DSF) from different drug-loaded fiber devices in PBS (pH: 7.4, 37 °C). (**c**) In vivo antitumor recurrence and metastasis inhibition analysis. Orthotopic tumor images and pulmonary metastasis images of 4T1-Luc tumor-bearing mice at different times. Lung metastasis fluorescence images were taken ex vivo after the mice were sacrificed on day 42. (**d**) SEM images of the fiber devices (* P < 0.05 and ** P < 0.01). Reprinted with permission from ref. [62]. Copyright 2020 John Wiley and Sons.

Multiple chemotherapeutic drug treatments help to prevent postoperative recurrence and metastasis of breast cancers, but traditional implantable devices are not capable of this [61]. Li et al. developed an implantable hierarchically structured ultrafine fiber device with time-programmed drug release of doxorubicin hydrochloride (DOX-HCL) to kill residual tumor cells and metalloproteinases-2 (MMP-2) inhibitor disulfiram (DSF) to prevent metastasis [62]. The implantable device is composed of the inner pipeline of a spinneret, which contained a polyethylene glycol (PEG) solution mixed with DOX-HCL, and the outer pipeline was filled with a poly(D,L-lactic acid) (PLA) solution mixed with MMP-2 inhibitor DSF. High water absorption of PEG caused the chambers of the fiber device to swell and rupture, releasing a high dose of DOX-HCL in a short period; meanwhile, MMP-2 inhibitor DSF had a slower release rate with the degradation of the fiber matrix. Devices with a varying mass of PEG (PEG-200, PEG-400, PEG-1000, and PEG-2000) were fabricated, and a faster DOX release rate was observed with the implants consisting of PEG with higher Mw (Figure 4a) with no significant difference in the release behavior of DSF (Figure 4b). Cell viability of 51.7% on day 7 indicated DOX-HCL was released at a suitable speed, and anti-recurrence and antimetastatic properties of the implants with the varying mass of PEG were assessed in vivo on tumor-bearing Balb/c mice up to 42 days. The intravenous injection group and the intratumoral injection group of the free drugs were considered as the controls. The best anti-recurrence and antimetastatic effects were observed in the mice treated with PEG-1000 (Figure 4c). Moreover, the group did not develop lung metastasis, and the recurrence tumor size was 80 times smaller than that by the injection administration. For in vivo study of tumor recurrence and metastasis, the orthotopic breast cancer model was established by injection of 1×10^6^ autologous fluorescence of luciferase gene-transfected 4T1 (4T1-Luc) cells in the gland fat pad of female Balb/c mice. The results validate that implantable device with multidrug release systems have the most significant capacity to prevent tumor recurrence and metastasis.

The work by Lei et al. concentrated on an injectable hydrogel based on poly(ethylene glycol)–poly(3-caprolactone)–poly(ethylene glycol) (PEG–PCL–PEG, PECE) loaded with PTX to prevent locoregional recurrence of 4T1 breast cancer in a mouse model [63]. Female Balb/c mice weighing 20 ± 2 g were used here. All the mice were housed at a temperature of 20–22 °C, relative humidity of 50–60%, and 12 h light–dark cycle. Free access to food and water was allowed. All the animals were in quarantine for a week before treatment. A consistent drug-release rate was obtained due to the sol–gel transition of the gel, and half of the PTX was released in the first 20 days. The recurrence rate was significantly decreased for the mice treated with a PTX-loaded PECE hydrogel. Only one mouse out of 11 developed locoregional tumor recurrence, which was significantly delayed to the 18^th^ day after tumor resection. Moreover, a moist environment provided by a large amount of water content in the PECE hydrogel facilitated fast wound healing. The tensile strength of the wound increased significantly after seven-day treatment.

### 3.2. Nanocomposite-Based Implantable Device

The efficacy of a porous silica–calcium phosphate nanocomposite (SCPC) as a new 5-FU delivery system was evaluated in vitro and in vivo [64]. SCPC has an excellent ability to tailor its dissolution rate, porosity, and physical form. The enhanced adsorption of 5-FU is due to the large surface area of the silica-rich sample. The pore size of SCPC’s unique porous structure can be adjusted by modifying the Si content in SCPC. The nanopores act as protective pockets for the adsorbed drug molecules. At the same time, the micropores enable continuous release of the drug by fluid exchange from the bulk of the SCPC scaffold to the surrounding tissues/cells. Two samples of the drug delivery device were fabricated by varying the Si content: SCPC50 (19.5% SiO_2_) and SCPC75 (32.9% SiO_2_). Each SCPC ceramic contained 37.9 ± 0.001 mg of 5-FU per g of SCPC50 and 40.2±0.001 mg of 5-FU perg of SCPC75, respectively. Male mice (Balb/c), following an acclimatization period, were injected subcutaneously with 2 × 10^5^ syngeneic 4T1 mammary tumor cells in the mammary fat pad in this work. Both samples with or without 5-FU were exposed to 4T1 mammary murine tumor cells to determine the toxicity and bioactivity of SCPC/5-FU hybrids. Cell numbers of 4T1 incubated with the samples without 5-FU were similar to the control, which indicates the biocompatibility of SCPC. A significant drop was observed in cell numbers of 4T1 incubated with both SCPC samples loaded with 5-FU; 4.7% of the adsorbed drug was released in an initial burst within 24 h. Afterwards, the drug was released at a sustained rate for up to 32 days. Subcutaneous implantations of SCPC 75–5-FU into mice showed a significant decrease in tumor volume (75% reduction) and tumor mass compared with the control sample (SCPC 75 without 5-FU).

**Figure 5.**
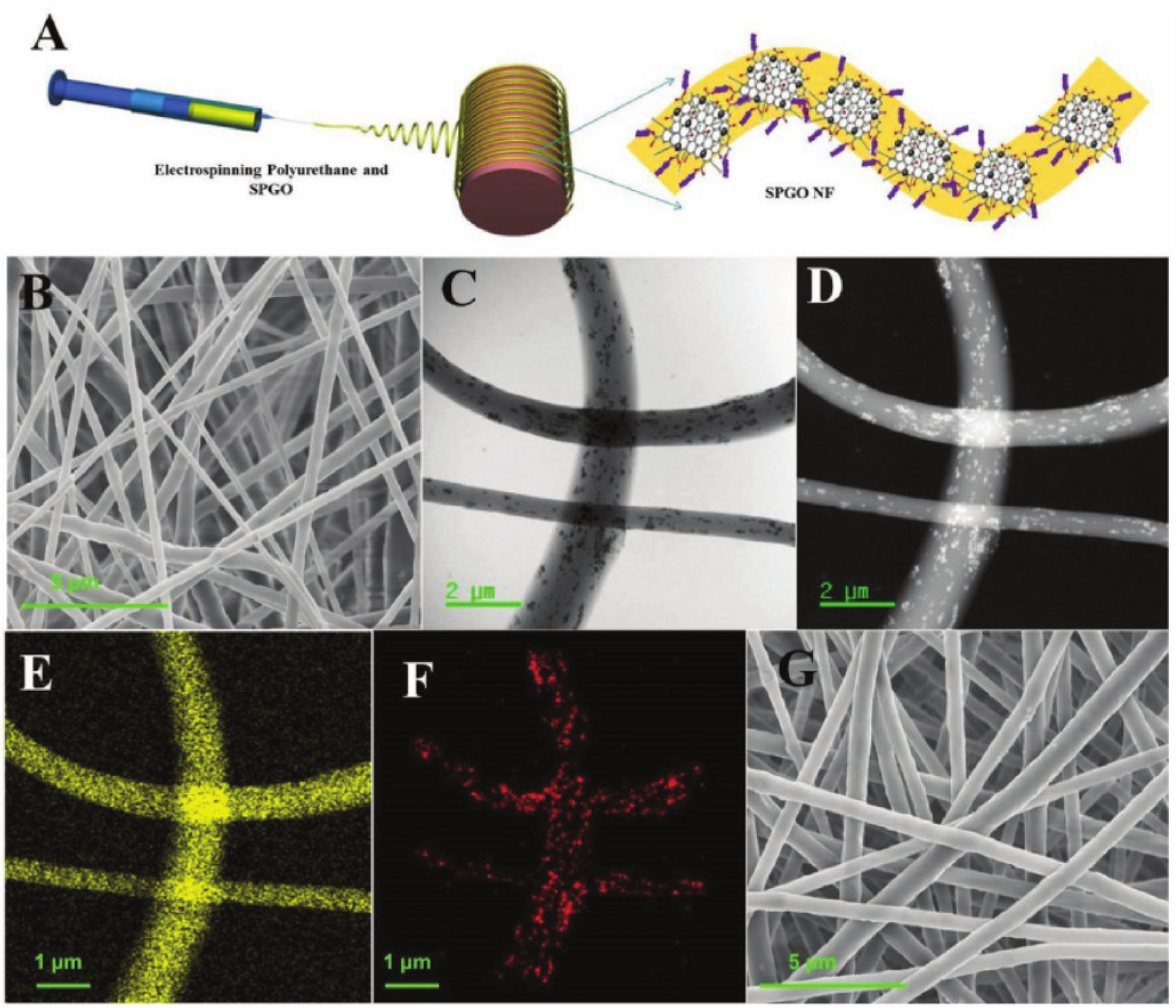
(**A**) Fabrication of the superparamagnetic graphene oxide (SPGO) NF. (**B**) FESEM image of the SPGO NF. (**C**,**D**) Brightfield and darkfield TEM images of the SPGO NF showing the uniform distribution of SPGO inside the NF matrix. (**E**) Element mappings of carbon. (**F**) Element mappings of iron. (**G**) FESEM image of the SPGO DOX NF. Reprinted with permission from ref. [65]. Copyright 2018 John Wiley and Sons.

An implantable anticancer device (IAD) with the functionality to prevent LRR of breast cancer as well as enhancement of breast reconstruction during/after therapy was proposed [65]. Superparamagnetic graphene oxide (SPGO) was fabricated by incorporating superparamagnetic iron oxide nanoparticles (SPION) with nanographene oxide [65,66]. DOX was conjugated with the resultant SPGO nanocomposite, then incorporated into a flexible and biocompatible polyurethane (PU) nanofiber matrix (SPGO DOX NF) (Figure 5). The average size of the resultant nanocomposite was ≈200 nm [65]. For the present study, 3T3L1 preadipocytes and MCF7 human breast cancer cell lines were used. The drug was released by the following two processes: diffusion-based and via pH, which acts as a triggering stimulus. A drug release study was carried out at two different pH levels: 5 and 7.4. At pH 5, the SPGO DOX NF showed a sustained DOX release (≈50%) for over 660 days without any initial burst release. This tumor-specific prolonged DOX delivery confirms the maximum efficacy of SPGO DOX NF as a postsurgical anticancer drug delivery device. Additionally, cyclic hyperthermia was applied for the inhibition of LRR. Nanoparticle sizes were kept at less than 20 nm for better hyperthermia performances [67]. The efficacy of the IAD to eliminate LRR was evaluated using MCF7 cell lines in three treatment scenarios: chemotherapy alone (SPGO DOX NF), hyperthermia (SPGO NF HT) alone, and combined HT and CT (SPGO DOX NF HT). The cells incubated with SPGO NFs and free DOX were considered as positive and negative controls, respectively. The cell proliferation index (CPI) of SPGO DOX NF HT reduced from 0.66 on day 1 to 0.19 on day 4. The high reduction rate of the CPI value confirms that combination treatment shows the most significant efficacy to terminate LRR. The nanofiber matrix in the IAD helps reconstruct poor breast cosmesis resulting from surgical treatment by supporting the lipofilling of the residual surgical cavity to induce the adipogenic process [65].

The selected notable works on implantable devices discussed in this paper are listed in Table 2.

**Figure 6.**
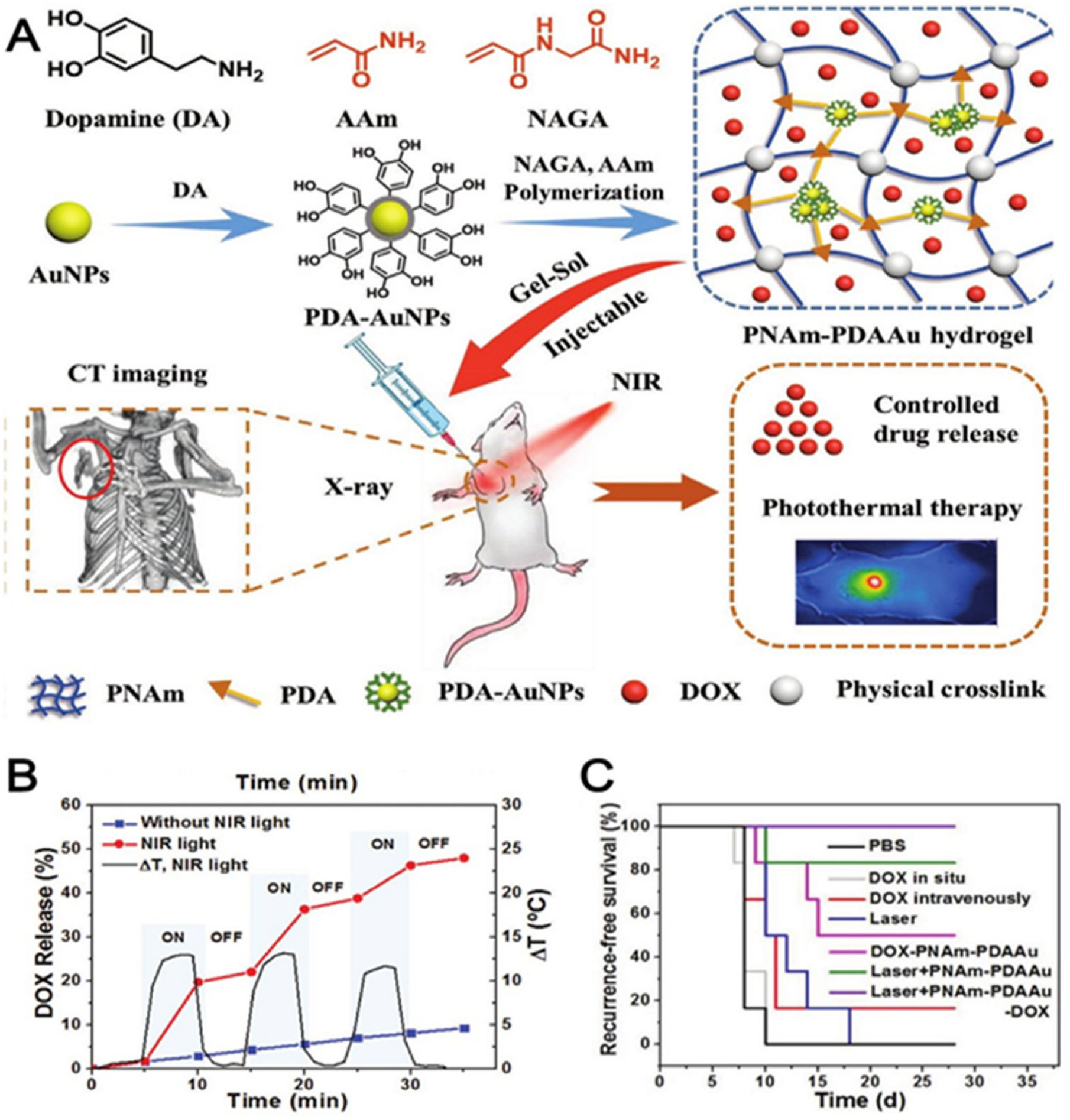
(**A**) PDA-AuNPs and nanocomposite PNAm–PDAAu hydrogel fabrication procedure and their theranostic application. (**B**) Photo triggered temperature increase and release of DOX from the PNAm–PDAAu hydrogel with and without NIR light irradiation. (**C**) In vivo breast cancer locoregional recurrence. PNAm—poly(N-acryloyl glycinamide-co-acrylamide); PDA—polydopamine. Reprinted with permission from ref. [68]. Copyright 2018 John Wiley and Sons.

Surgical removal of the whole breast or a significant portion being removed is considered one of the primary treatments for breast cancer. However, the high recurrence rate of breast cancer poses a significant risk for patient survival. Wu et al. designed a DOX-incorporated injectable thermoresponsive supramolecular poly(N-acryloyl glycinamide-co-acrylamide) (PNAm) hydrogel bearing polydopamine (PDA) with 20 nm thick coated-gold nanoparticles (AuNPs) due to its 808 nm wavelength absorption peak [68]. The sol–gel conversion of injected PNAm–PDAAu–DOX solution fills the breast cavity, and the application of near-infrared (NIR) light induces a photothermal effect (Figure 6A) which releases the drug as the polymer network is weakened. The weakened network-enabled drug to be released at a higher rate through diffusion (Figure 6B). In order to produce orthotopic primary tumors, the right thoracic mammary fat pad of each Balb/C mice out of six was injected with 10^6^ 4T1 cells in 100 μL PBS. No locoregional tumor recurrence was observed in the therapeutic group of mice treated with the PNAm–PDAAu–DOX hydrogel with laser irritations due to the controlled release and photothermal effect. The recurrence-free survival percentage was 100% for 30 days (Figure 6C).

## 4. Challenges of Clinical Translation of Implantable Devices

Over the last one-decade, significant progress was made towards understanding the implantable therapeutic devices and its interaction with the human immune system [69,70]. Human immune cells play a significant role in successful implantation of these devices and their function in the body for an extended period [71–74]. In this regard, mouse or rat models play a significant role in understanding this process, even though it is not quite similar to the human immune system. The majority of the implantable devices in cancer and breast cancer models were tested in mouse or humanized mouse models as the rat model has its own limitation [75–79]. In this regard, a humanized mouse model has provided a great solution to bridge the gap [80–82]. It is an immunodeficient mouse model that often reconstitutes human immune cells [83]. There are many different humanized mouse models that provide very specific answers with respect to how implantable devices may work in the human body [84–86]. They may also show how sustainable these devices will be inside the human body in the future based on the immune reaction to these devices in these mouse models, and often it also depends on the graft versus host disease (GVHD) reaction of that model [84]. However, since some breast cancers are not considered to be highly immunogenic, this phenomenon may help in installing implantable devices in humans and therapy execution [87]. In addition, a humanized mouse model can also provide a detailed scenario of how implantable device-based immune therapy might work in some types of breast cancers [88–91]. Considering the invasive nature of breast cancer, any implantable devices need careful evaluation of possible causes of the human immune reaction due to those devices. In this regard, the majority of the approaches used a patient-derived xenograft (PDX) model which provides a similar effect as a humanized model [92,93]. Therefore, implantation of a human xenograft in a humanized mouse model or immunocompetent mouse model can provide detailed understanding of macrophage biology and immune regulation that may occur in humans [94–96]. However, these models need further validation as different types of breast cancer can have different immune reactions and different mouse models may provide different information about the disease and therapeutic strategies. In this regard, humanized mouse models have provided great tools to answer some of the questions regarding development of more advanced implantable devices. This implantable drug delivery system (IDDS) or similar treatment devices are expected to be in constant contact with the surrounding host tissue or body fluid of human or other test animal models. Therefore, such a device has to meet the United States FDA’s criteria for biocompatibility before it gets clinical approval. The challenge of making a biocompatible implant for humans is understanding the human immune response to implants, also referred as foreign body reaction (FBR) [97]. In addition, most of the implantable devices for human therapeutic purposes are also evaluated based on their pharmaceutical purpose and regulatory prospects as they may have drugs, biological products (such as antibodies, etc.); each component of the device itself will have an individual effect on the human immune system [98]. In order to bring the device from bench to bedside, it has to go through the discovery phase in all three categories as mentioned above, and then it will be evaluated for its safety and durability in preclinical and clinical developmental phase. A successful candidate then proceeds through the market approval process and enters market or large-scale production for human trials in the framework of future monitoring [98–100]. Overall, the process of animal to clinical translation may take several months to years depending on the purpose of the implantable device.

## 5. Conclusions and Future Prospects

Conventional treatment for breast cancer can show noticeable results due to the advancement in drug therapeutics, but the probability of recurrence is high. Treatments such as chemotherapy and radiation therapy encounter obstacles in treating the disease efficiently. For example, local chemotherapy causes systemic toxicity or damages other healthy organs as the chemotherapeutic drugs travel through the bloodstream into the affected region. A similar scenario is observed in the case of radiation therapy. Patients going through these kinds of treatments experience severe side effects. They harm the quality of life for breast cancer patients. Extirpation of the breast during the surgical removal of the cancerous tumor can have grave consequences for the patient’s mental health. The physiological stress and time-consuming hospitalization can sometimes lead patients to terminate the overall treatment. Implantable drug delivery devices are designed to overcome these hurdles through local delivery of therapeutic agents directly to the tumor site. The implantable devices discussed here use nanomaterials, biopolymers, or a combination of the two. Drugs released from these devices can be controlled with an external stimulus such as pH or temperature, giving precise control over the release kinetics, thus enhancing the overall efficacy of the treatment procedure. In addition, targeted drug delivery reduces the adverse side effects of therapeutic drugs. Furthermore, the devices deliver various anticancer drugs and can use combination therapy, such as chemotherapeutic drugs in conjunction with hyperthermia which has shown noticeable results in preventing breast cancer recurrence in animal models. The biocompatible polymers used in the implantable devices significantly improve the effectiveness of surgical interventions. Additionally, polymer matrices or scaffolds are used in the implantable devices, which can help with breast reconstruction and wound healing. All these benefits provide great potential for developing multifunctional implantable devices for efficient and harmless cancer therapy. Even with all the advantageous properties, treating breast cancer with implantable drug delivery devices is still far from being clinically implemented. All the results discussed here are about the devices being implemented in xenografts and immunocompromised animals which are not a suitable representation of the human immune system. Therefore, further studies are needed with better animal models such as immunocompetent animals and humanized mice, at least with respect to elements of the human immune system. More research needs to be carried out on treating a metastatic disease or breast cancer that has spread beyond the breast and lymph nodes using implantable drug delivery devices. This can be achieved by using an approach which combines both local delivery and systemic delivery of drugs. The treatment of breast cancer without surgical removal of the breast can be another area in which implantable drug delivery can be used. Much more research is needed in the area of material selection. Acquiring the suitable materials for the implantable device, which would cause no adverse effect on the human body, would not react with the drug, making the removal of the device easier, or making the resultant device easily absorbed in the human body is still a challenge. However, the explicit control over release kinetics of the drugs, targeted drug delivery, increased drug efficiency, and breast reconstruction make implantable drug delivery devices a predominant therapeutic platform for treating breast cancer. Moreover, recent advances in artificial intelligence (AI) techniques can also be integrated into implantable targeted drug delivery devices. Implantable devices with integrated sensors can provide feedback to external controllers, which in turn regulate the drug release by accelerating the release rate. Further integration with the Internet of Things (IoT), wireless communication, and cloud technologies can allow efficient and effective communication with caregivers, immediately inform them in the case of unexpected developments, and generate long-term analysis in correlation with other relevant data.

## Figures and Tables

**Table 1.** Implantable devices for cancer therapy.

Implantable Device	Type of Cancer Treated	Reference
Osmotic system-based implantable device	Prostate cancer	[30]
β-lapachone-containing polymer implants (millirods)	Prostate cancer	[31]
Magnetically controlled drug delivery MEMS device	Prostate cancer	[32]
Biocompatible poly(glycerol monostearate-co-e-caprolactone) polymer film-loaded PTX	Lewis lung carcinoma	[33]
Polymeric delivery platform made up of poly(glycerol monostearate-co-ε-caprolactone) films loaded with HCPT	Lewis lung carcinoma	[34]
Polyester nanofibers of the PLGA-PLA-PCL nanofiber implant	Brain gliomas	[35]
Smart hyperthermia nanofibrous scaffolds consisting of the *N*-isopropylacrylamide and *N*-hydroxymethylacrylamide polymers	Skin cancer	[38]

PTX—paclitaxel; HCPT—10-hydroxycamptothecin.

**Table 2.** Types of implantable devices for breast cancer therapy.

Implantable Devices	Notable Features	Reference
PLGA incorporated with CS-DOX	Inhibit 4T1 breast tumor growth and metastasis, thus increasing the survival rate from 60 days to 115 days	[55]
Intelligent 3D PLGA, gelatin, and chitosan scaffold loaded with 5-FU and DOX	Good blood-clotting ability helps with wound healing, and no damage was observed in the liver, spleen, and kidney tissues	[56]
Scaffolds were made up of PLGA and immobilized with 5-FU and NVP-BEZ235	Decrease cancer cell viability to less than 30% after 7 days and tumor growth after 4 weeks	[57]
DOX-loaded silk films	MDA-MB-231 viability and tumor weight significantly decreased	[58]
DTX-loaded PDLLA nanofibers	Prolonged delivery and a sufficient local cytotoxic drug preventing local tumor recurrence	[59]
Hierarchically structured fibers with hydrophilic internal chambers (containing PEG and DOX-HCl) and hydrophobic fiber matrix (containing PLA and DSF)	Tumors in the three mice out of five treated with the implant completely disappeared and in the other mice, they decreased by 80%	[62]
Injectable hydrogel based on PEG-PCL-PEG, PECE loaded with PTX	Recurrence rate in vivo was significantly decreased and fast wound healing was observed	[63]
SCPC nanocomposite	No significant growth of 4T1 breast tumors was seen after 40 days	[64]
SPGO nanocomposite incorporated with nanofiber matrix PU and drug DOX	Proliferation percentage of 3T3L1 cell lines decreased from around 60% on day 1 to less than 20% on day 4	[65]
PNAm hydrogel bearing PDA-coated AuNPs and DOX	No locoregional tumor recurrence was observed and overall survivability rate increased	[68]

CS-DOX—chitosan-loaded doxorubicin; DTX—docetaxel; PDLLA—poly-D,L-lactide; PLA—poly(lactic acid); DSF—disulfiram; SCPC—silica–calcium phosphate nanocomposite; SPGO—superparamagnetic graphene oxide; PU—polyurethane; PNAm—poly(N-acryloyl glycinamide-co-acrylamide); PDA—polydopamine.
